# Low-threshold surface-emitting colloidal quantum-dot circular Bragg laser array

**DOI:** 10.1038/s41377-024-01714-9

**Published:** 2025-01-07

**Authors:** Yangzhi Tan, Yitong Huang, Dan Wu, Yunjun Wang, Xiao Wei Sun, Hoi Wai Choi, Kai Wang

**Affiliations:** 1 State Key Laboratory of Optical Fiber and Cable Manufacture Technology, Institute of Nanoscience and Applications, Department of Electrical and Electronic Engineering, Southern University of Science and Technology, Shenzhen, China; 2 Department of Electrical and Electronic Engineering, The University of Hong Kong, Hong Kong, China; 3 College of New Materials and New Energies, Shenzhen Technology University, Shenzhen, China; 4 Suzhou Xingshuo Nanotech Co., Ltd. (Mesolight), Suzhou, China

**Keywords:** Semiconductor lasers, Quantum dots

## Abstract

Colloidal quantum dots (CQDs) are attractive gain media due to their wavelength-tunability and low optical gain threshold. Consequently, CQD lasers, especially the surface-emitting ones, are promising candidates for display, sensing and communication. However, it remains challenging to achieve a low-threshold surface-emitting CQD laser array with high stability and integration density. For this purpose, it is necessary to combine the improvement of CQD material and laser cavity. Here, we have developed high-quality CQD material with core/interlayer/graded shell structure to achieve a low gain threshold and high stability. Subsequently, surface-emitting lasers based on CQD-integrated circular Bragg resonator (CBR) have been achieved, wherein the near-unity mode confinement factor (Γ of 89%) and high Purcell factor of 22.7 attributed to the strong field confinement of CBR enable a low lasing threshold of 17 μJ cm^−^^2^, which is 70% lower than that (56 μJ cm^−^^2^) of CQD vertical-cavity surface-emitting laser. Benefiting from the high quality of CQD material and laser cavity, the CQD CBR laser is capable of continuous stable operation for 1000 hours (corresponding to 3.63 × 10^8^ pulses) at room temperature. This performance is the best among solution-processed lasers composed of nanocrystals. Moreover, the miniaturized mode volume in CBR allows the integration of CQD lasers with an unprecedentedly high density above 2100 pixels per inch. Overall, the proposed low-threshold, stable and compactly integrated CQD CBR laser array would advance the development of CQD laser for practical applications.

## Introduction

Colloidal quantum dots (CQDs) are emerging semiconductor nanocrystals with attractive optoelectronic characteristics, including their wide-range wavelength-tunability, solution-processibility and low optical gain threshold^1^. These properties make CQDs promising for developing non-epitaxial laser diodes compatible with easily scalable and cost-effective fabrication and integration techniques^2^.

Over the recent decades, substantial efforts have been dedicated to the development of CQD lasers, leading to significant improvements in their lasing performance and integration technology. On one hand, in the aspect of material engineering, CQDs with continuously graded (cg) core-shell structure (e.g., CdSe/Cd_x_Zn_1-x_Se) have been synthesized, wherein the carrier confinement potential was smoothened to suppress the Auger recombination^3^. Furthermore, these cg-CQDs were charged to reduce the ground-state absorption, leading to the demonstration of near-zero-threshold optical gain (<*N*_*th,gain*_> = 0.02, corresponding to 0.02 exciton per dot on average)^4^ and sub-single-exciton lasing with unprecedentedly low threshold of 2.1 μJ cm^−^^2^ (<*N*_*th,las*_> = 0.31)^5^. As the available optical gain and lasing threshold of CQDs are approaching the theoretical limit (<*N*_*th*_> = 0), further reduction of the threshold through CQD material engineering poses a challenge. On the other hand, various laser cavities embedded with CQDs have been developed, such as distributed feedback (DFB) resonator^6^, vertical-cavity surface-emitting laser (VCSEL)^7^, whispering gallery mode (WGM)^8^, and plasmonic lattice^9^. Among these, the surface-emitting lasers are generally more desirable in many applications such as displays, sensing and optical interconnect compared to the edge-emitting (or unidirectional-emitting) ones due to their capability for two-dimensional integration, relatively narrower divergence angle and lower power consumption^10–12^. In practical applications, surface-emitting lasers are commonly employed in the form of two-dimensional array with high integration density instead of individual devices. However, although there have been many reports of integrated CQD lasers, most of them are in WGM^13–21^. In contrast, the integrated surface-emitting CQD laser array is rarely reported^22,23^, with a limited integration density below 300 pixels-per-inch (PPI).

Several challenges complicate the realization of low-threshold surface-emitting CQD laser array with high integration density. First, the field confinement ability of typical surface-emitting resonators such as VCSEL and DFB is relatively weak, leading to a large mode volume (*V*) in the cavity that limits the laser integration density. *V* is defined as^24^:

1 $$V=\frac{{\int }_{V}\varepsilon (r){|E(r)|}^{2}dV}{\max (\varepsilon (r){|E(r)|}^{2})}$$

where *ε*, *E*(*r*), *dV* are the dielectric permittivity, the local electric field intensity in three-dimensional space and the volume element, respectively. Typically, the Purcell factor (*F*_*P*_), which measures the ratio of spontaneous emission rate (*R*_*r,cavity*_) inside a cavity to that in free space (*R*_*r,0*_), is negatively correlated to the *V*. The expression is written as^25^:

2 $${F}_{P}=\frac{{R }_{r,cavity}}{{R }_{r,0}}=\frac{3}{4{\pi }^{2}}{\left(\frac{\lambda }{n}\right)}^{3}\frac{Q}{V}$$

where *λ*, *n* and *Q* represent the vacuum wavelength, medium refractive index and cavity quality factor, respectively. Generally, a larger *F*_*P*_ contributes to reducing the lasing threshold (*P*_*th,las*_) and may even lead to a threshold-less laser in principle^26,27^. Therefore, it is necessary to enhance the field confinement in CQD-embedded resonator to achieve a higher *F*_*P*_ and consequently lower *P*_*th,las*_.

Second, in most surface-emitting CQD lasers, the CQDs only serve as the gain media integrated inside the cavity but not the constituent part of the cavity. Consequently, the mode confinement factor (Γ) that measures the fraction of the electric field energy confined to the active region (Γ *=* *∫*_*active*_|*E(r)*|^2^*dV*/*∫*_*cavity*_|*E(r)*|^2^*dV*), is typically small in those cavities. For example, due to the limited distribution of CQD in VCSEL cavity, the Γ is generally below 50%^7,28^. As the modal gain (<*g*>) is proportional to Γ (<*g*> = Γ*g*, where *g* is the material gain), the small Γ would impede the low-threshold lasing action in CQD surface-emitting laser.

In this work, we have addressed these challenges by employing a surface-emitting CQD-integrated circular Bragg resonator (CBR) with strong field confinement that allows low-threshold lasing and compact integration of the surface-emitting laser array. Compared with the VCSEL composed of dielectric DBRs and sandwiched CQD gain medium, the CQDs in CBR laser not only act as the gain medium, but also as the high-index component of the CBR cavity. Consequently, the Γ in CQD CBR laser has been increased from 39% to 89% compared with the CQD VCSEL. Furthermore, as the CBR is essentially based on a two-dimensional radially symmetric photonic crystal (PhC) structure that supports Bragg reflection at all azimuthal angles, it offers a superior spatial field confinement than the VCSEL that is based on one-dimensional PhC structure^29,30^. As a result, the *V* of about 3*(*λ*/*n*)^3^ in CQD CBR laser is significantly reduced by 92% compared with that (~38*(*λ*/*n*)^3^) in CQD VCSEL, leading to an enhanced peak *F*_*P*_ from 3.7 to 22.7 at resonant wavelength. Benefiting from the improved Γ and *F*_*P*_, the CQD CBR laser exhibits a *P*_*th,las*_ of ~17 μJ cm^−^^2^ under 300-ps pulsed excitation, while the corresponding figure of the CQD VCSEL is ~56 μJ cm^−^^2^. Such a low *P*_*th,las*_ along with the high-quality CQD material contributes to the excellent long-term working stability of the CBR laser. The operational lifespan of the CQD CBR laser exceeded 1000 hours, equivalent to 3.63 × 10^8^ lasing pulses, making it the longest among the reported solution-processed lasers composed of nanocrystals. Moreover, the strongly confined field distribution in CQD CBR laser facilitates its compact two-dimensional integration. A CQD CBR laser array with an integration density of 2100 PPI has been achieved. Notably, this integration density is the highest among the reported surface-emitting CQD lasers to our knowledge. In conclusion, the low-threshold, high-stability, ease-of-integration characteristics of the surface-emitting CQD CBR laser would be beneficial for the further development of practical non-epitaxial (e.g., organics, perovskites and CQDs) lasers.

## Results

### Design and characterization of CQDs

**Fig. 1:**
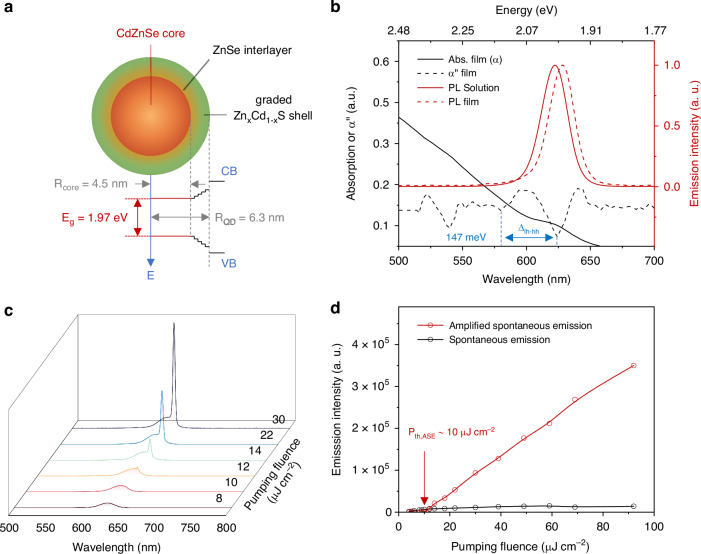
Structure and photoluminescence characteristics of the CdZnSe/ZnSe/Zn_x_Cd_1-x_S CQDs. **a** The cross-section and internal band diagram of the CdZnSe/ZnSe/Zn_x_Cd_1-x_S CQDs. **b** The PL spectra of CdZnSe/ZnSe/Zn_x_Cd_1-x_S CQDs in solution (red solid line) and film (red dashed line); the linear absorption (*α*, black solid line) spectrum of CQDs film and its second-order derivative (*α*”, black dashed line). **c** The emission spectra of CQDs film at various *P* from 8 μJ cm^−2^ to 30 μJ cm^−2^. **d** The pumping fluence-dependent intensity of spontaneous emission (in black) and ASE (in red) of CQDs film

The CQDs used in this work possess a type-I CdZnSe/ZnSe/Zn_x_Cd_1-x_S core/interlayer/graded shell structure (see Fig. 1a). The ZnSe interlayer with intermediate lattice constants at the core-shell interface was designed to relax compressive lattice strain during shell formation and promote conformal and uniform shell development^31,32^. Besides, the introduced compositional gradients in the CQD structure serve to smoothen the carrier confinement potential and subsequently suppress Auger recombination by inhibiting the intra-band transitions involved in the dissipation of the electron-hole recombination energy^2,3^. According to the theoretical analysis by Efros et al.^33^ and experimental demonstration by Klimov et al.^3,34^, the continuously graded CQDs (cg-CQDs) are highly desirable for light amplification applications due to their high biexciton quantum yield (QY_XX_) and sub-single-exciton optical gain, which originate from the suppressed Auger decay in these CQDs. Moreover, the mean diameter of CQDs is designed to ~12.6 nm (see Supplementary Fig. S1a), which is smaller than that of cg-CQDs (typically ~19 nm) and comparable to that reported as “compact” cg-CQDs^35^, to enable a relatively high packing density in the CQD film that allows a high material gain while maintaining the effective suppression of Auger decay in CdZnSe/ZnSe/Zn_x_Cd_1-x_S CQDs.

The photoluminescence (PL) spectra of CdZnSe/ZnSe/Zn_x_Cd_1-x_S CQDs in solution and film are given in Fig. 1b, respectively, showing a peak emission wavelength of 629 nm and a full-width-at-half-maximum (FWHM) of 23 nm in CQDs film. The QY of the CQDs solution and film under low pumping fluence is 96% and 80%, respectively. The linear absorption (*α*) spectrum of CQDs film and its second-order derivative (d^2^*α*/d*λ*^2^, marked as *α*″) are also shown in Fig. 1b, in which three transition paths including the band-edge 1S_e_–1S_hh_ transition (marked as 1S at *λ* = 624 nm), 1S_e_–1S_lh_ (1S’ at *λ* = 581 nm) and 1 P_e_–1P_hh_ (1P at *λ* = 546 nm) transition can be clearly distinguished. The detailed information of band-edge states (1 P_e_, 1S_e_, 1S_hh_, 1S_lh_ and 1P_hh_) and three transition paths (1S, 1S’ and 1P) of CdZnSe/ZnSe/Zn_x_Cd_1-x_S CQDs can be found in Supplementary Fig. S1b. The light-heavy hole splitting (*Δ*_*lh-hh*_), which measures the energy difference between 1S and 1S’ transitions, is about 147 meV for CdZnSe/ZnSe/Zn_x_Cd_1-x_S CQDs. This value is more than twice of those reported in cg-CQDs^22,35^ (56 meV) and asymmetrically strained CQDs^36^ (up to 62 meV) and six times the thermal energy (*k*_*B*_*T* = 25.9 meV at *T* = 300 K, where *k*_*B*_ is the Boltzmann constant) at room temperature (RT). This contrast helps improve the thermal stability of the lasing characteristics by inhibiting the thermal-induced depopulation of the 1S band-edge states in CQDs^37^.

To characterize the amplified spontaneous emission (ASE) characteristics of the CdZnSe/ZnSe/Zn_x_Cd_1-x_S CQDs, we used a 355 nm pulsed laser with pulse width of 300 ps, repetition rate of 100 Hz as the pumping source capable of exciting the CQDs to their multi-excitonic states. The emission spectra of CQDs at various pumping fluence are illustrated in Fig. 1c. Under low pumping fluence, the CQDs exhibit a single spontaneous emission peak at 629 nm with a FWHM of 23 nm. When increasing the pumping fluence to above 10 μJ cm^−^^2^, another emission peak at about 639 nm with an FWHM of 4 nm appears, indicating the onset of ASE at 1S band. The red-shift from spontaneous emission to ASE indicates the attractive exciton-exciton (X-X) interaction in CQDs, which typically occurred in type I CQDs^37^. The pumping fluence-dependent intensity of spontaneous emission and ASE is illustrated in Fig. 1d, showing that the threshold of 1S-band ASE (*P*_*th,1S ASE*_) is about 10 μJ cm^−^^2^. Besides, it can be observed that the intensity of 1S-band ASE increases dramatically with the increased pumping fluence, while the intensity of spontaneous emission tends to saturate when pumping fluence is above 40 μJ cm^−^^2^. Furthermore, when the pumping fluence is further increased, another emission peak at 600 nm occurs (see Supplementary Fig. S1c), indicating the ASE action at 1S’ band when CQDs are excited to bi-excitonic states. The X–X interaction energy (*Δ*_*XX*_), which measures the Coulomb interactions between excitons in CQDs, can be calculated by the energy gap between two ASE bands (*Δ*_*XX*_ = *E*_*1S’ ASE*_—*E*_*1S ASE*_) that is about 129 meV. The theoretical gain threshold in terms of <*N*_*th,gain*_> can be calculated as <*N*_*th,gain*_> = 2/[3-exp(-*Δ*_*XX*_^2^/*γ*^2^)], where *γ* represents the FWHM (in eV) of spontaneous emission of CQDs at RT^38^. When adopting a *Δ*_*XX*_ of 129 meV and *γ* of 72 meV for CdZnSe/ZnSe/Zn_x_Cd_1-x_S CQDs, we can obtain a <*N*_*th,gain*_> = 0.676, which is even below the theoretical value of <*N*_*th,gain*_> = 1 for neutral type-I cg-CQDs^37^. The low-threshold optical gain characteristic of the CdZnSe/ZnSe/Zn_x_Cd_1-x_S CQDs would significantly facilitate the low-threshold lasing action in the following research.

### Cavity design and analysis

**Fig. 2:**
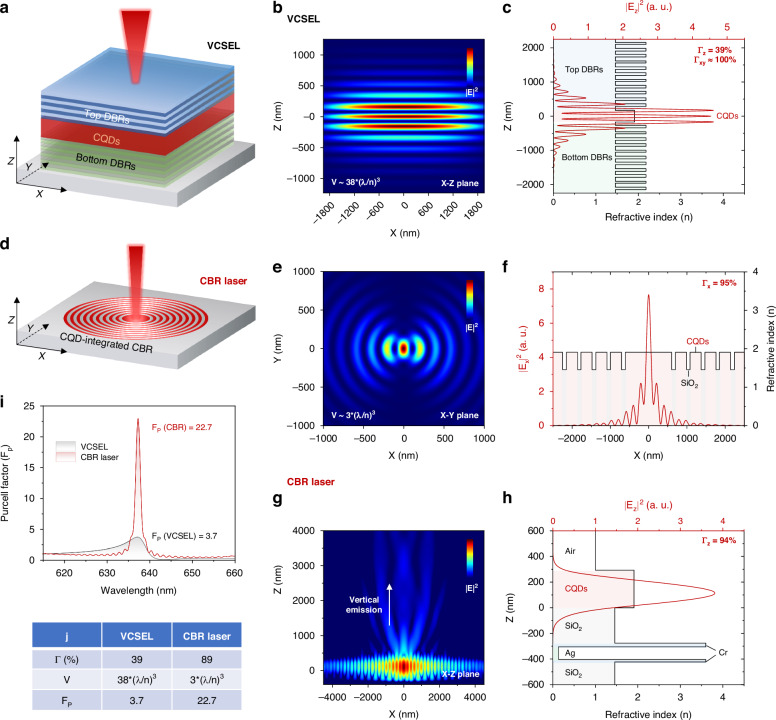
Simulation of the CQD VCSEL and CBR laser. **a** The structure of CQD VCSEL. The squared electric field intensity (|*E*|^2^) distribution in **b** X-Z plane and **c** along Z axis within the VCSEL. The *V*, Γ of VCSEL are calculated to be ~38*(*λ*/*n*)^3^ and 39%, respectively. **d** The structure of CQD CBR laser. The |*E*|^2^ distribution in **e** X-Y plane and **f** along *Y* axis within the CBR laser. The *V*, Γ_x_ (or Γ_xy_) of CBR laser are calculated to be about 3*(*λ*/*n*)^3^ and 95%, respectively. **g** The |*E*|^2^ distribution in X-Z plane within the CBR laser, where |*E*|^2^ is in the logarithmic scale. Vertical emission from the CBR center can be observed. **h** The |*E*|^2^ distribution along the Z-axis within the CBR laser. The Γ_z_ of CBR laser is ~94%. **i** The *λ*-dependent *F*_*P*_ in VCSEL and CBR laser. **j** The table summarizes the Γ, V, and peak *F*_*P*_ in VCSEL and CBR laser

The structure of CQD VCSEL is illustrated in Fig. 2a, where the CQDs are sandwiched between top- and bottom-DBRs consisting of 16 pairs of Ta_2_O_5_/SiO_2_ fabricated by plasma-assisted e-beam evaporation. The refractive indices of Ta_2_O_5_ and SiO_2_, CQDs are characterized by ellipsometer and are provided in Supplementary Fig. S2a, b, e. According to the simulation and characterization, the DBRs exhibit a reflectivity as high as 99.9% from 578 nm to 692 nm (see Supplementary Fig. S2c), indicating a high quality of their fabrication. The resonant wavelength *λ* of the VCSEL is set to 637 nm, and its cavity length is designed as *λ*/*n* to support single-mode lasing at 637 nm. The squared electric field intensity (|*E*|^2^) of the resonant mode (*λ* = 637 nm) within the VCSEL cavity is illustrated in Fig. 2b, by which we can calculate a *V* of ~38*(*λ*/*n*)^3^ in VCSEL as per Eq. 1. It is worth mentioning that the smallest reported *V* for VCSEL is ~10*(*λ*/*n*)^3^, which was achieved by employing a micropillar VCSEL structure that supports lateral mode confinement in addition to the longitudinal one. Figure 2c shows the longitudinal field distribution (|*E*_*z*_|^2^) in VCSEL, where the longitudinal Γ (Γ_z_) is calculated to be 39%. As the dimension of our VCSEL is in the millimeter range, its lateral Γ (Γ_xy_) can be approximated as near-unity^12^, leading to a Γ close to 39%.

In contrast to the VCSEL with a vertically stacked sandwich structure, the CQD CBR laser possesses a pancake-like structure consisting of CQDs-integrated circular Bragg gratings and reflective substrate (see Fig. 2d). The planar cavity structure of CBR laser offers several advantages over traditional DBR-based surface-emitting cavities, including a simplified fabrication process that avoids time-consuming micrometer-thick DBR deposition and facilitates large-scale array formation using established patterning techniques such as lithography and nanoimprinting. The structural parameters of CBR laser are well optimized to support Bragg reflection at azimuthal directions, thereby confining the optical field to the center of CBR. The |*E*|^2^ distribution in the X-Y plane within the CBR laser is illustrated in Fig. 2e, showing that the field energy has been well confined to a small region near the CBR center, leading to a *V* of about 3*(*λ*/*n*)^3^. Figure 2f shows the |*E*|^2^ distribution along the X-axis. Benefiting from the wide distribution of the gain medium—CQDs in the gain-integrated CBR structure, the Γ_x_ (or Γ_xy_ due to the symmetry of CBR) of CBR laser is as high as 95%. The |*E*|^2^ distribution in the X-Z plane is presented in Fig. 2g. When setting the |*E*|^2^ in logarithmic scale, it can be observed that some of the energy is escaping from the CBR center, indicating the vertical surface emission of CBR laser. The vertical emission from the CBR laser is primarily due to the stronger field confinement in the X-Y plane than that in the Z-axis. It should be noted that the vertical emission from a CBR can be both upward and downward. To achieve unidirectional top emission, a bottom silver mirror is necessary. Besides, as the refractive index *n* of CQD (*n*_*CQD*_ = 1.91) is higher than those of air (*n*_*air*_ = 1) and SiO_2_ (*n*_*SiO2*_ = 1.46), the CBR laser exhibits a Γ_z_ of about 94%. The overall Γ in CBR laser is calculated to be 89% by Γ *=* *∫*_*active*_|*E(r)*|^2^*dV*/*∫*_*cavity*_|*E(r)*|^2^*dV*.

The *λ*-dependent *F*_*P*_ in VCSEL and CBR laser is shown in Fig. 2i, wherein the CBR laser exhibits a peak *F*_*P*_ of 22.7 at *λ* = 637 nm, which is about six times the magnitude of that of VCSEL (3.7). It should be noted that the *F*_*P*_ of 22.7 is observed at the centre of the CBR, where the |*E*|^2^ is at its maximum. The high *F*_*P*_ of CBR laser could be attributed to the strong localized |*E*|^2^ inside the CBR enabled by its excellent field confinement ability. The Γ, *V* and peak *F*_*P*_ of VCSEL and CBR laser are summarized in Fig. 2j, showing that the Γ and *V* in CBR laser have been increased by 128% and decreased by 92% compared with the VCSEL, respectively, along with a 514% increased peak *F*_*P*_. The enhanced mode confinement in CBR laser is significant for improving its lasing performance, which will be discussed in the later section.

### Fabrication of CQD CBR laser array and VCSEL

**Fig. 3:**
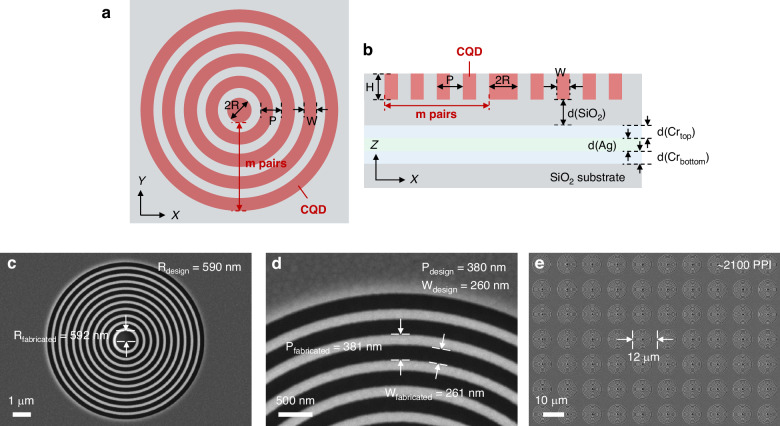
Structure and fabrication of the CQD CBR laser array. The **a** top and **b** side view of the structure of CQD CBR laser. **c**–**e** SEM images of the CBR unit and array. The outer diameter of each CBR laser unit is ~9 μm. With a designed dot pitch of 12 μm as depicted in Fig. 3e, we achieved a pixel density of ~2100 PPI calculated by pixel density (in PPI) = 1 inch/dot pitch = 25400 μm/dot pitch

The entire structure of CQD CBR laser is illustrated in Fig. 3a, b, where detailed structural parameters are marked out. From the top view as shown in Fig. 3a, the structural parameters include the radius of the inner CQD disk (*R*), the period of circular Bragg grating (*P*), the width of CQD in a period (*W*) and the *m* of CBR. From the side view as shown in Fig. 3b, the CBR structure is based on a SiO_2_ substrate. A thin silver film serves as the reflector and is sandwiched by CBR and substrate to enable unidirectional top emission from the CBR laser. Two chromium layers are introduced as the bonding layer between silver and SiO_2_. The refractive indices of the relevant materials can be found in Supplementary Fig. S2a, b, d–f. All parameters are well optimized to enable a high *F*_*P*_ at the desired wavelength close to the first ASE peak of CQDs. Considering the inevitable structural deviation between design and processing, we have designed three sets of structural parameters with *F*_*P*_ peak wavelengths of 637 nm, 640 nm, and 643 nm, respectively, to accommodate fabrication errors (see Supplementary Fig. S3a–d). The detailed fabrication process of the CQD CBR laser based on electron beam lithography (EBL) can be found in Supplementary Fig. S4. According to the scanning electron microscopy images given in Fig. 3c, d, the fabricated CBR structure aligns well with the design. For example, the designed *R*, *P* and *W* of the CBR with an *F*_*P*_ peak at 637 are 590 nm, 380 nm and 260 nm, while those of the fabricated sample are 592 nm, 381 nm and 261 nm, respectively. The high precision of fabrication facilitates the achievement of desired lasing characteristics in CQD CBR lasers. Furthermore, a CBR array with a dot pitch of 12 μm and a high integration density of 2100 PPI has been successfully fabricated as shown in Fig. 3e. It is worth mentioning that this integration density is the highest among the reported surface-emitting CQD lasers with planar cavity structures (e. g., DFB, bound-state-in-the-continuum, PhC and plasmonic lattice cavity) as shown in Supplementary Table 1. The implementation of such a high integration density could be attributed to the small *V* in CBR, which enables us to miniaturize it under the premise of great mode confinement as discussed in the former Section. Moreover, benefiting from the high-quality fabrication based on EBL, the CBR units in array exhibit great uniformity, which is crucial for achieving near-single-mode lasing from the laser array. Besides, the cavity of CQD VCSEL has also been carefully controlled to achieve single-mode lasing at desired wavelength.

### Lasing characteristics of CQD CBR laser and VCSEL

**Fig. 4:**
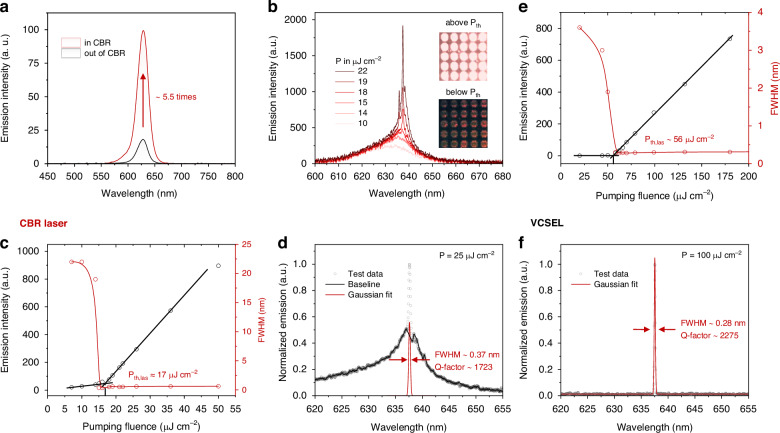
Lasing characteristics of VCSEL and CBR laser. **a** PL spectra of CQDs in and out of CBR under low pumping intensity. **b** PL spectra of CQD CBR laser under different pumping intensities from 10 μJ cm^−^^2^ to 22 μJ cm^−^^2^. The inset photographs show the CBR laser array operating below and above its lasing threshold. **c** Pumping fluence-dependent emission intensity and FWHM of CQD CBR laser. **d** Emission spectrum of CQD CBR laser under pumping fluence of 25 μJ cm^−^^2^ and its Gaussian fitting. **e** Pumping fluence-dependent emission intensity and FWHM of CQD VCSEL. **f** Emission spectrum of CQD VCSEL under pumping fluence of 100 μJ cm^−^^2^ and its Gaussian fitting

First, the PL of CQDs in and out of CBR under low pumping intensity has been characterized (see Fig. 4a), showing that the PL intensity of CQDs on CBR is 5.5 times higher than that off CBR. This PL enhancement has confirmed a strong positive Purcell effect as predicted, although the PL enhancement is not as significant as the simulated *F*_*P*_ peak of 22.7. It should be noted that the *F*_*P*_ of 22.7 is only observed at the center of the CBR, where the field intensity is at its maximum. However, in the characterization, we collected the PL signal from CQDs in anywhere within the CBR array. Therefore, the PL enhancement factor of 5.5 could be approximately considered as an average *F*_*P*_ within the CBR. We have conducted a simulation on the *F*_*P*_ of dipoles placed at different positions within the CBR cavity (see Supplementary Fig. S5a, b). The results show a simulated average *F*_*P*_ peak of ~6.7, which is close to the experimental result. Additionally, the FWHM of the CBR-coupled spontaneous emission spectrum is observed to be broader than that of the *F*_*P*_ spectrum (Fig. 2i). This broadening can be attributed to two factors: (i) the *β* factor, which quantifies the fraction of spontaneous emission coupled into the cavity mode, is estimated to be approximately 0.13 in the CBR cavity (see Supplementary Fig. S6b). Although this value is significantly higher than that of the VCSEL (5 × 10^−^^3^, see Supplementary Fig. S6a), the spontaneous emission spectrum within the CBR cavity may still exhibit a broader linewidth compared to the narrow cavity mode, resembling the PL spectrum in free space; (ii) the macroscopic nature of our PL measurements, which integrate the emission from multiple CBR units. Slight variations in the mode positions across different units contribute to the overall broadening of the PL spectrum. These factors collectively result in the observed broader FWHM of the CBR-coupled spontaneous emission spectrum relative to the *F*_*P*_ spectrum.

The lasing characteristics of the CQD CBR laser have been characterized using a 355 nm laser pumping source with pulse width of 300 ps. The PL characterization setup is illustrated in Supplementary Fig. S7.The pumping fluence-dependent PL spectra are given in Fig. 4b. A distinct transition from spontaneous emission to lasing at 638 nm is observed as the pumping fluence is increased from 10 μJ cm^−^^2^ to 22 μJ cm^−^^2^. The photographs of the CQD CBR laser array also show that the emission intensity is significantly improved above the lasing threshold *P*_*th,las*_. As illustrated in Fig. 4c, the FWHM of the CBR laser emission experiences a sudden decrease from 20 nm to about 0.40 nm, accompanied by a super-linear increase in the emission intensity near the lasing threshold *P*_*th,las*_ of about 17 μJ cm^−^^2^. The *P*_*th,las*_ is among the lowest reported for surface-emitting CQD lasers with planar cavity structure and is comparable to those pumped by fs-laser (see Supplementary Table 1). In comparison, the *P*_*th,las*_ of CQD VCSEL is determined to be about 56 μJ cm^−^^2^ as shown in Fig. 4e. We have tested 10 samples for each CBR laser and VCSEL to verify the reproducibility (see Supplementary Fig. S8). The results show that the average *P*_*th,las*_ of CBR laser and VCSEL are 21.9 μJ cm^−^^2^ and 63.6 μJ cm^−^^2^, respectively. The significant reduction in *P*_*th,las*_ from VCSEL to CBR laser could be attributed to the stronger Purcell effect and larger Γ in CBR laser as discussed above. Besides, the fitting of lasing spectrum of CBR laser gives an emission FWHM of about 0.37 nm, corresponding to a quality factor (Q-factor) of 1723 (see Fig. 4d). The Q-factor of CBR laser is lower than the fabricated CQD VCSEL (2275, see Fig. 4f) but higher than the reported CQD VCSELs (up to about 1300) in literature^7,28,39^, confirming the relatively high quality of the developed CBR cavity. The primary limitation hindering further improvement in the Q-factor of the CBR laser could be attributed to increased scattering losses arising from imperfections in the optical structure and non-uniformity in the CQDs film^11,12^, particularly when the field energy is highly localized in the CBR. This trade-off between *V* and Q-factor could be mitigated by improving the CQD film quality and/or designing CBR structures that effectively decouple the optical field antinodes from the structural boundary. Besides, it is noteworthy that due to the large spot size (~120 × 160 μm^2^) of our PL setup, it inevitably captures spontaneous emission from CQDs located outside the CBR, contributing to background noise in the PL spectra (see Fig. 4b, d). This issue can be probably addressed by acquiring PL spectra from localized micro-regions.

**Fig. 5:**
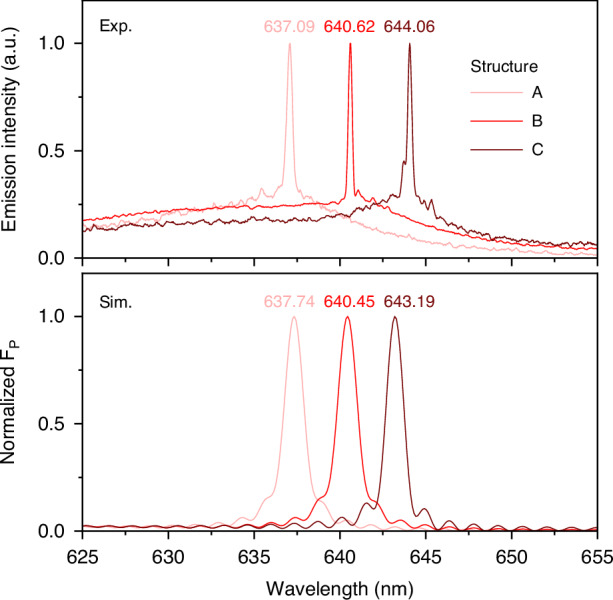
Tunability of the CQD CBR laser. The upper figure shows the characterized emission spectra of CBR lasers with three sets of structure parameters (marked in A, B and C). The lower gives the normalized *λ*-dependent *F*_*P*_ of three CBR lasers

Moreover, the resonant wavelength in CBR laser can be precisely adjusted by controlling its structural parameters (e.g., *R*, *P,* and *W*), allowing us to tune its lasing wavelength (*λ*_*las*_) and integrate CBR lasers with various *λ*_*las*_ monolithically. As depicted in the upper of Fig. 5, CBR lasers with tunable *λ*_*las*_ from 637.09 nm to 644.06 nm have been achieved. The tunable range is limited by the bandwidth of optical gain (as reflected in the ASE spectrum) of CQDs. The lower figure in Fig. 5 illustrates the simulated *F*_*P*_ spectra of CBR lasers with different sets of structure parameters (marked as A, B, C), showing a great coincidence between the experimental and simulated results as the deviation of emission peak wavelength is within 1 nm.

**Fig. 6:**
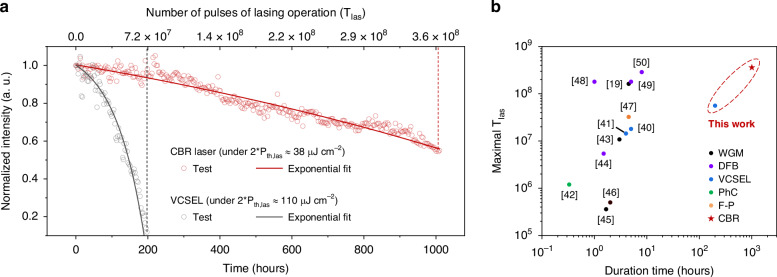
Long-term stability of lasing from CQD CBR laser and VCSEL. **a** The evolution of lasing intensity of CQD CBR laser (in red) and VCSEL (in black) under 100 Hz, 300 ps excitation for 1007 hours and 200 hours, respectively. **b** The comparison of reported maximal *T*_*las*_ of solution-processed nanocrystal (CQD and colloidal quantum well) lasers^18,19,40–50^ with various cavity structures and those of CQD CBR laser and VCSEL in this work

Benefiting from the high quality of the developed CQDs material in terms of high QY and low-threshold optical gain, as well as the low *P*_*th,las*_ of CQD CBR laser enabled by the strong mode confinement within it as discussed above, the CQD CBR laser exhibits a great long-term stability of lasing operation. Figure 6a illustrates the evolution of the lasing intensity of CQD CBR laser (in red) and VCSEL (in black) under 100 Hz ps-pulsed excitation for 1007 hours and 200 hours, respectively. The lasers were encapsulated with UV glue to isolate them from ambient water and oxygen. The pumping fluence for CBR laser and VCSEL were kept at 38 μJ cm^−^^2^ and 110 μJ cm^−^^2^, respectively, which are about twice their *P*_*th,las*_. For CQD VCSEL, its lasing intensity drops to about 10% of its initial state after 200-hour excitation, corresponding to 7.2 × 10^7^ times of pulses of lasing operation (*T*_*las*_). When compared to CQD VCSELs^40,41^ with reported stability data (maximum *T*_*las*_ from 1.44 × 10^7^ to 1.6 × 10^7^, time duration from 4 to 5 hours), our VCSEL exhibited significantly enhanced stability despite being excited at a higher *P* of 110 μJ cm^−^^2^ (@300 ps pulse width) than the reported values of 3.5–4.0 μJ cm^−^^2^ (@100 fs pulse width). We attribute this superior stability to the high-quality core/interlayer/graded shell structure of our CQDs, which ensures a high PLQY of 96% and effectively suppresses non-radiative Auger recombination. Additionally, the large *Δ*_*lh-hh*_ of 147 meV in our CQDs, approximately six times the thermal energy of 25.9 meV at RT, effectively inhibits thermal-induced depopulation of the 1S band-edge states, further enhancing the thermal stability of the CQD lasing characteristics.

For the CQD CBR laser, its lasing intensity remains above 55% after 1007 hours (or *T*_*las*_ = 3.63 × 10^8^) continuous operation at room temperature. This significant improvement in lasing stability from VCSEL to CBR laser could be attributed to the reduced pumping fluence required to achieve intense lasing action. It should be mentioned that the *T*_*las*_ of 3.63 × 10^8^ is the best among those reported for solution-processed nanocrystal (CQD and colloidal quantum well) lasers^18,19,40–50^ as shown in Fig. 6b. Detailed information can be found in Supplementary Table 2. Besides, this is the first report of solution-processed nanocrystal laser that undergoes stable lasing operation for over 1000 hours. The excellent lasing characteristics and stability evidence the effectiveness of the CQD material engineering as well as the high quality of the developed CBR laser cavity. We believe the combination of improved material and laser cavity could promote the further development of CQD laser towards electrically driven lasing.

## Discussion

In this study, low-threshold surface-emitting CQD laser array with excellent operational stability and high integration density has been demonstrated by combining engineered high-quality CQD material with a CBR cavity with strong field confinement capability. First, CdZnSe/ZnSe/Zn_x_Cd_1-x_S CQDs with high efficiency, great stability and sub-single-exciton gain threshold characteristics have been developed, facilitating the achievement of high-performance CQD lasers. Subsequently, CQDs were integrated into a two-dimensional PhC structure named CBR, whose strong mode confinement ability results in a large Γ of 89% and a high *F*_*P*_ of 22.7, which are superior to those in CQD VCSEL (Γ = 39% and *F*_*P*_ = 3.7). In consequence, the developed CQD CBR laser exhibits a low lasing threshold of 17 μJ cm^−^^2^, which is 70% lower than that (56 μJ cm^−^^2^) of CQD VCSEL. The high quality of the cavity and CQD material of the CBR laser contribute to its excellent stability throughout the 1000-hour operation, which is the best among its nanocrystal-based counterparts. Furthermore, the small *V* in CBR laser facilitates its compact integration with exceptionally high density above 2100 PPI. The combination of these exceptional characteristics in the proposed CQD CBR laser array paves the way for significant advancements in solution-processed lasers for various practical applications, including displays, sensing and communication.

## Materials and methods

### Synthesis of CQDs

The detailed synthesis process of CdZnSe/ZnSe/Zn_x_Cd_1-x_S CQDs can be found in Supplementary Note 1.

### Fabrication of the CQD CBR laser array

The fabrication process is illustrated in Supplementary Fig. S4.

### PL characterization

For ASE and laser characterization, a diode-pumped solid-state pulsed laser, MPL-FN-355 from CNI, emitting at 355 nm with a pulse width of 300 ps and a repetition rate of 100 Hz was used as the pump source. The pumping fluence is adjusted by two laser power attenuators (Optogama LPA-M 355 nm) in series. The signal was collected using a ×50 lens (GU Optics, ×50 M PLAN APO NIR) with 0.45 NA and a working distance of 20.06 mm. The spectrometers coupled to the system are Ocean Optics USB2000+ and AvaSpec ULS4096CL EVO. The PL characterization setup is illustrated in Supplementary Fig. S7.

### Design and simulation

The design and simulation of the CBR laser and VCSEL in this work were accomplished using Lumerical FDTD.

## Supplementary information

Supplementary Information for Low-threshold surface-emitting colloidal quantum-dot circular Bragg laser array

## Data Availability

The data that support the findings of this study are available from the corresponding authors upon reasonable request.
