# Supplementary material for: Low-threshold surface-emitting colloidal quantum-dot circular Bragg laser array
Source: Light Sci Appl. 2025 Jan 7;14:36. doi: 10.1038/s41377-024-01714-9 (PMC11704271; doi:10.1038/s41377-024-01714-9)
Supplement: Supplementary file 1 — Supplementary Information for Low-threshold surface-emitting colloidal quantum-dot circular Bragg laser array [file 41377_2024_1714_MOESM1_ESM.pdf]

Supplementary Information for

Low-threshold surface-emitting colloidal quantum-dot circular Bragg laser array

Yangzhi Tan *et al.*

Corresponding authors.

Email: wudan@sztu.edu.cn, sunxw@sustech.edu.cn, hwchoi@hku.hk, wangk@sustech.edu.cn

Supplementary Note 1. Synthesis process of the CdZnSe/ZnSe/$Zn_xCd_{1-x}S$ CQDs

Precursor Preparation

Selenium Precursor (Se-TOP):

1. Under an inert atmosphere within a glovebox, 20 mmol of Se powder and 40 mL of trioctylphosphine (TOP) were weighed and loaded into a 100 mL three-necked flask.
2. The mixture was heated to 150°C with continuous stirring until complete dissolution of the Se powder was achieved.
3. The solution was then allowed to cool down to room temperature for subsequent use.

Sulfur Precursor (S-TOP):

1. In a glovebox, weigh 20 mmol of S powder and 40 ml of TOP into a 100 ml three-necked flask.
2. The mixture was heated to 120°C with stirring until complete dissolution of the S powder was observed.
3. The solution was then cooled down to room temperature for further use.

Cadmium Precursor ($Cd(OA)_2$):

1. Weigh 1 mmol of CdO, 10 ml of oleic acid, and 40 ml of ODE into a 100 ml three-necked flask.
2. The flask was purged with argon gas before raising the temperature to 210°C. The mixture was heated until a clear solution was obtained.
3. The $Cd(OA)_2$ precursor solution was then cooled down to room temperature for later use.

Synthesis Procedure:

1. A 100 mL three-necked flask was charged with 0.2 mmol of CdO, 4 mmol of zinc acetate ($(CH_3COO)_2Zn$), 5 mL of oleic acid, and 15 mL of ODE under an inert atmosphere.
2. The flask was then degassed under vacuum at 120°C for 30 minutes to remove any residual oxygen.
3. After degassing, the flask was purged with argon gas, and the reaction temperature was raised to 280°C.
4. Subsequently, 0.5 mmol of Se-TOP solution was swiftly injected into the flask, and the reaction mixture was maintained at 280°C for 60 minutes.
5. The reaction temperature was then reduced to 260°C, and simultaneously, 0.8 mmol of $Cd(OA)_2$ precursor solution and 3 mmol of S-TOP solution were co-injected over a period of 1 hour. This step allows for the formation of the core/shell/alloyed-shell structure.
6. After the co-injection step, the reaction mixture was cooled down to 80°C. The synthesized CQDs were then purified twice using a mixture of hexane and ethanol to remove any unreacted precursors or impurities.

Notes:

- All manipulations throughout the synthesis process were conducted under an inert atmosphere to prevent oxidation of the precursors and ensure the quality of the final product.
- The reaction temperature and time should be carefully controlled to ensure the formation of the desired product.
- A thorough purification process is essential to eliminate any residual impurities that could potentially affect the optical and electronic properties of the final CQD product.

Supplementary Figure 1. Characterization of the CdZnSe/ZnSe/$Zn_xCd_{1-x}S$ CQDs

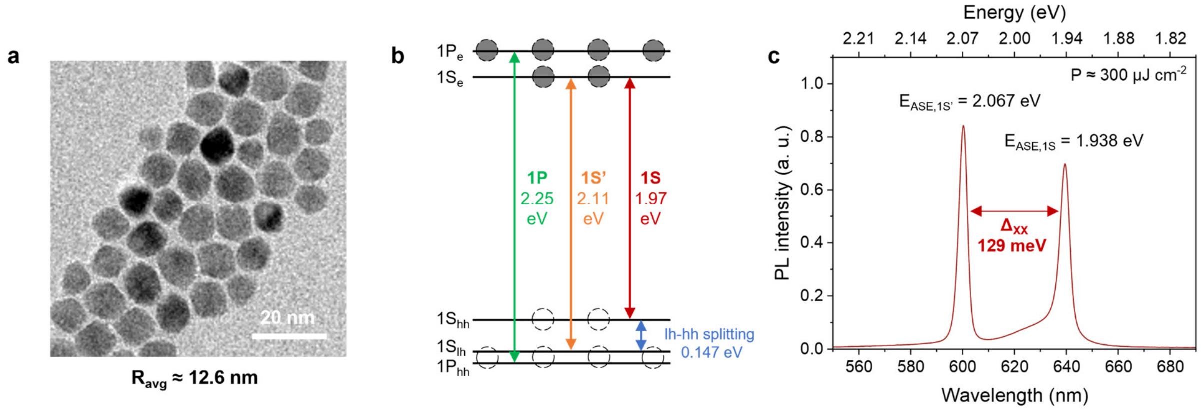

(a) The transmission electron microscopy (TEM) images of the QDs used in this work, the scale bar is 20 nm. The average diameter of the CQDs is about 12.6 nm.

(b) The derived band-edge states ($1P_e$, $1S_e$, $1S_{hh}$, $1S_{lh}$ and $1P_{hh}$) and three transition paths (1S, 1S' and 1P) of CQDs. The light-heavy hole splitting ($\Delta_{lh\text{-}hh}$), which measures the energy difference between 1S and 1S' transitions, is about 147 meV.

(b) The two-band amplified spontaneous emission (ASE) spectrum of CQDs excited under a high pumping fluence (P) of about 300 μJ cm^{-2}. The X–X interaction energy (Δ_{XX}), which measures the Coulomb interactions between excitons in CQDs, can be calculated by the energy gap between two ASE bands ($\Delta_{XX} = E_{1S'ASE} - E_{1S\,ASE}$) that is about 129 meV.

Supplementary Figure 2. Refractive indices of various materials and characterization of DBRs

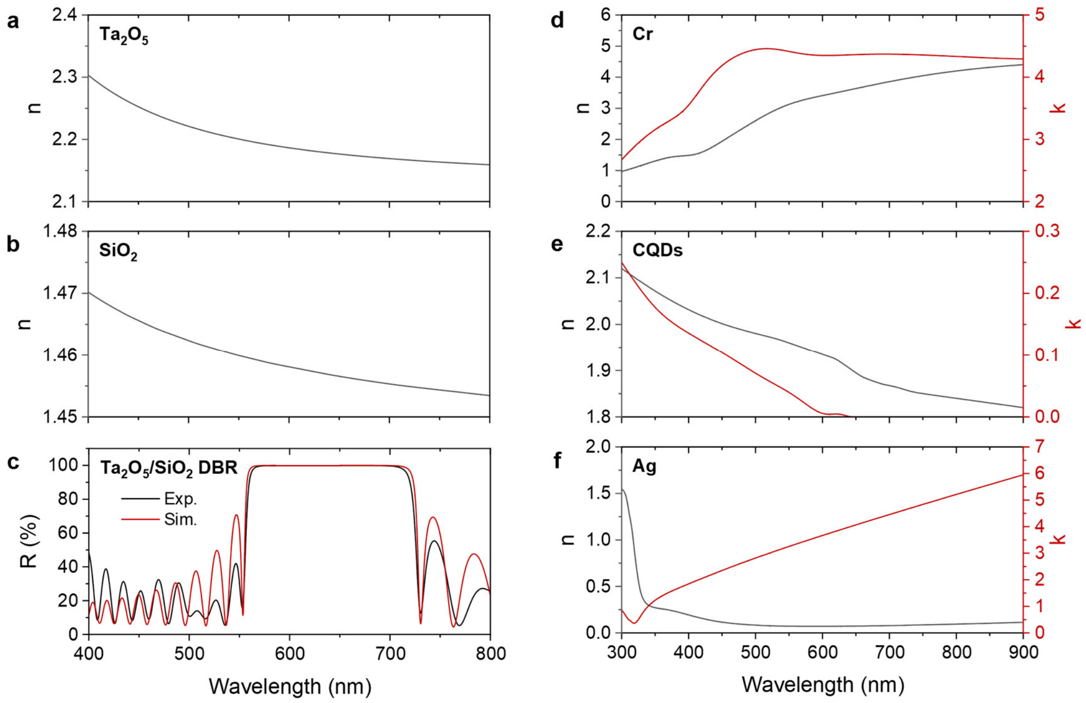

(a, b) The real (n) part of the refractive indices of Ta_2O_5 and SiO_2.

(c) The simulated and characterized reflectance spectrum of the 16-pair DBRs composed of Ta_2O_5 and SiO_2. Each pair contains 73 nm Ta_2O_5 and 109 nm SiO_2. The targeted center wavelength of maximum reflectance of normal incidence is about 635 nm, calculated by $\lambda = 4n_{Ta2O5}d_{Ta2O5} = 4n_{SiO2}d_{SiO2}$.

(d-f) The n and imaginary (k) part of the refractive indices of Cr, CQDs and Ag.

Supplementary Figure 3. The detailed structure parameters of CQD CBR laser

a

CBR Structure	A	B	C
R (nm)	590	595	600
P (nm)	380	385	390
W (nm)	260	265	270
H (nm)		270	
$d(SiO_2)$ (nm)		280	
$d(Cr_{top})$ (nm)		30	
d(Ag) (nm)		100	
$d(Cr_{bottom})$ (nm)		20	
m		10	
F_P peak wavelength (nm)	637	640	643
F_P maximum	22.7	22.8	29.6

b

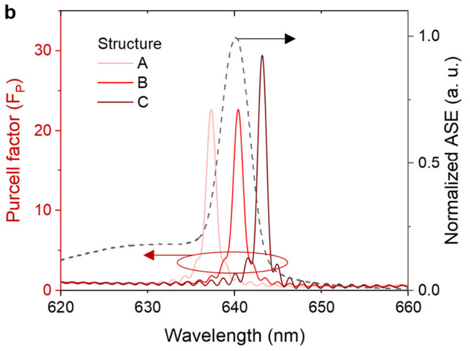

c

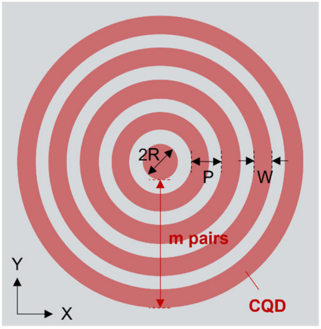

d

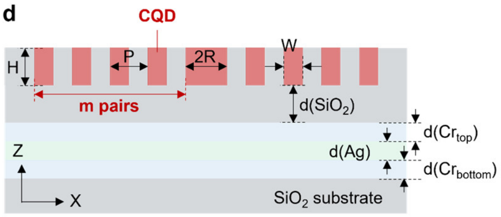

(a) The detailed structure parameters of three CBR lasers and the corresponding Purcell factor (F_P) peak and peak wavelength. *R*, *P*, and *W* are the radius of the inner disk, period and width of CQDs in a CBR period, respectively, as depicted in Fig. S3c. *H*, $d(SiO_2)$, $d(Cr_{top})$, *d(Ag)* and $d(Cr_{bottom})$ are the height of CBR structure, thickness of the SiO_2 interlayer, thickness of the top Cr bonding layer, thickness of the Ag layer and thickness of the bottom Cr bonding layer, respectively, as marked in Fig. S3d. m is the number of pairs of CBR.

(b) The wavelength-dependent F_P (in red) of CBR lasers with different structures (A, B, C) and the ASE spectrum (in dashed black) of CQD.

(c) and (d) are the top- and side-view of the CBR laser.

Supplementary Figure 4. Fabrication process of the CQD CBR laser

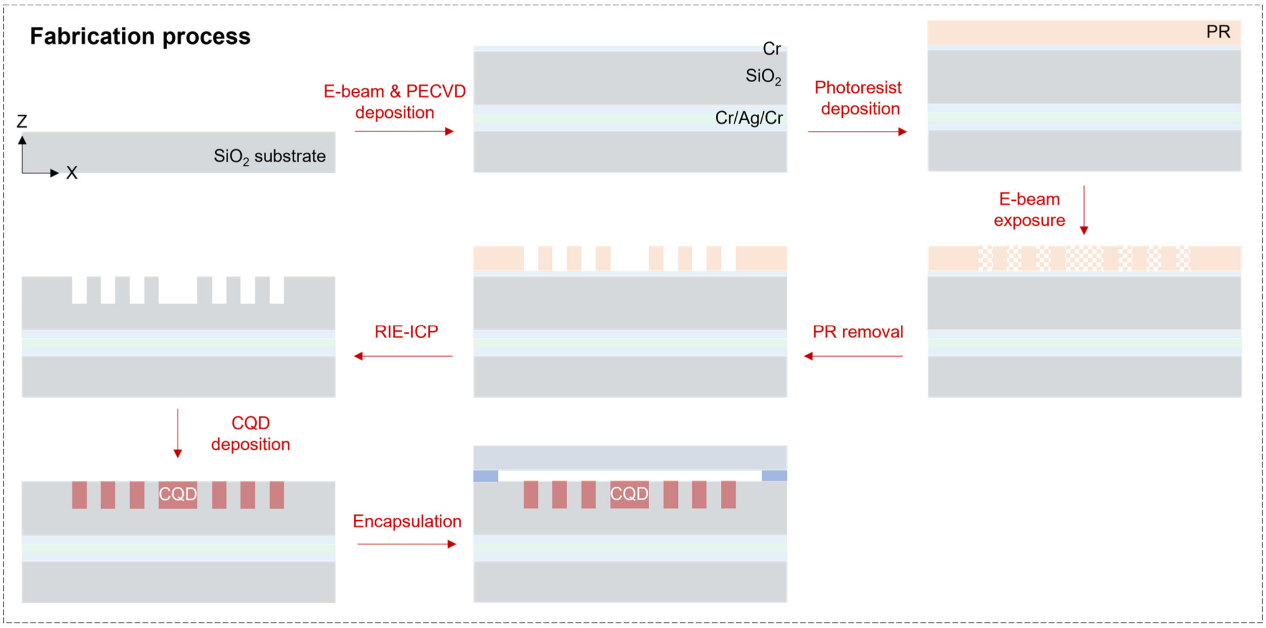

The provided image depicts the process of electron beam lithography (EBL)-based fabrication of the CQD CBR laser.

1. Substrate preparation: the process begins with a SiO_2 substrate, which was thoroughly cleaned to remove any contaminants that could affect the subsequent steps.

2. Thin film deposition: Cr and Ag thin films were deposited using electron-beam evaporation. A 550 nm SiO_2 layer was deposited by plasma enhanced chemical vapor deposition (PECVD).

3. Photoresist deposition: positive photoresist (PR) is deposited onto the Cr layer by spin-coating.

4. Electron beam exposure: PR is exposed to electron beam that defines the pattern of CBR.

5. Development: PR is selectively removed in the areas exposed to electron beam.

6. Etching and lift-off: sacrificial layers were removed using inductively coupled plasma reactive ion etching (ICP-RIE), leaving behind leaving behind the final SiO_2 cavity structure.

7. CQD deposition: CQDs are spin-coated onto the SiO_2 cavity structure to fill the gap in CBR structure. The fabrication of CQD CBR laser is completed.

8. Encapsulation: the device is encapsulated by UV glue to isolate it from moisture and oxygen in atmosphere. This step is crucial to ensure a great working stability of CQD CBR laser.

Supplementary Figure 5. Simulation of F_P at different positions in CBR

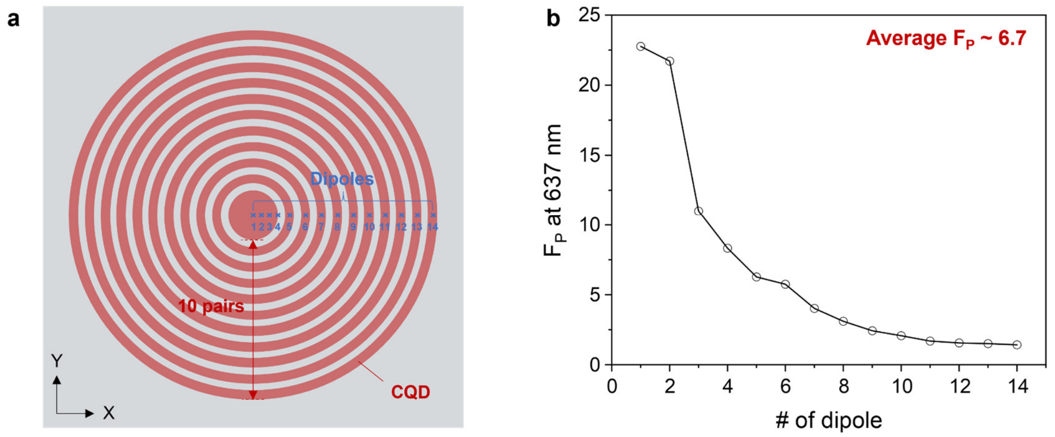

(a) The top view of CBR laser with structure A and F_P peak wavelength of 637 nm. Fourteen dipoles are placed at different positions in CBR.

(b) The F_P (at 637 nm) of different dipoles. The F_P decreases as the dipole position moves further away from the center of the CBR. The average F_P (at 637 nm) of these dipoles is about 6.7.

Supplementary Figure 6. Estimation of the spontaneous emission coupling factor *β* in CBR laser and VCSEL

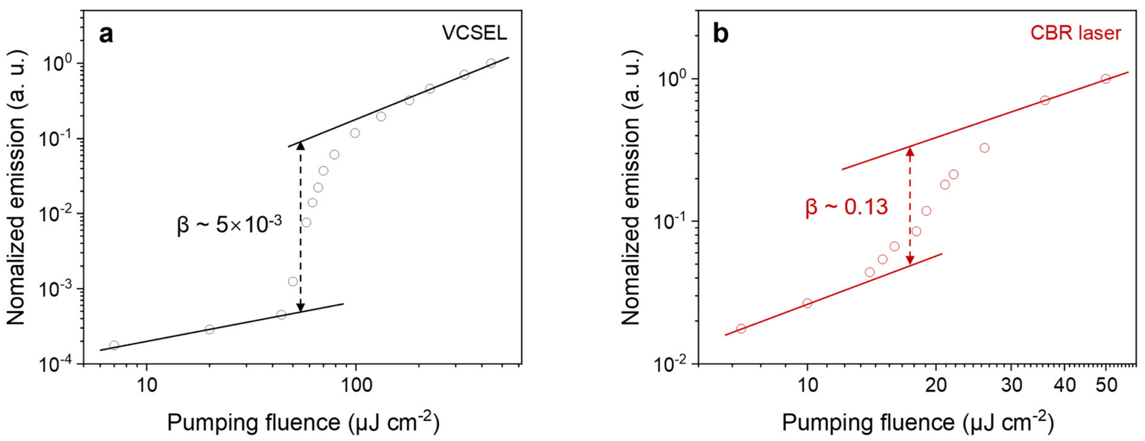

Pumping fluence-dependent emission intensity of (a) the CQD VCSEL and (b) the CQD CBR laser, plotted on log-log scales. The *β* factor, which quantifies the fraction of spontaneously emitted photons coupled into the cavity mode, was estimated to be 5×10^{-3} for the VCSEL and 0.13 for the CBR laser.

Supplementary Figure 7. Photoluminescence characterization setup

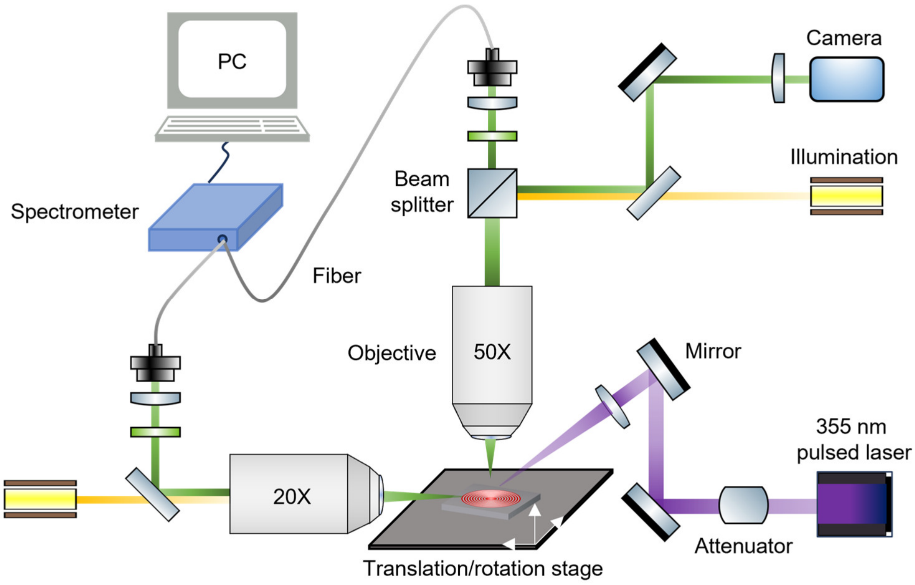

The structure of PL characterization setup. The objective in the Z-axis is the primary component for signal collection due to the relatively weak edge emission from the sample. The light collection spot size for this setup using 50× objective is approximately 120 × 160 μm^2. The numerical aperture (NA) of the 50× objective in the setup is 0.45, corresponding to a maximum collection angle of 26.7°.

Supplementary Figure 8. Statistics of lasing threshold ($P_{th,las}$) of CQD CBR laser and VCSEL

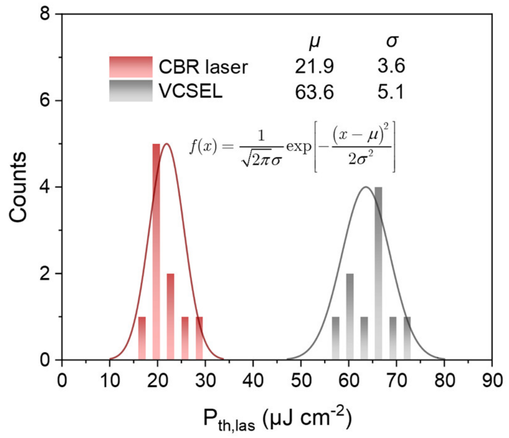

To evaluate the reproducibility and consistency of the fabricated CBR lasers and VCSELs, we performed $P_{th,las}$ measurements on ten CBR lasers and ten VCSELs. The statistics are depicted in the figure above. The data were fitted with Gaussian functions, revealing mean $P_{th,las}$ (μ) of 21.9 μJ cm^{-2} and 63.6 μJ cm^{-2} for CBR lasers and VCSELs, respectively. The corresponding standard deviations (σ) are 3.6 and 5.1, respectively.

Supplementary Table 1. Comparison among the reported surface-emitting CQD and colloidal quantum well (CQW) lasers with planar cavity structure

Pump source		Gain medium	Cavity structure	$P_{th,las}$ (μJ cm^{-2})	λ_{peak}, FWHM (nm)	Q-factor	Pixelation?	Reference
fs pulse	400 nm, 150 fs, 1 kHz	CdSe/CdZnS/ZnS CQDs	1D DFB	77	652-662, <1.00	> 650	No	*Advanced Functional Materials* 22, 337-344 (2011)
	450 nm, 340 fs, 1 kHz	CdSe/CdZnS CQDs	2D DFB	110	638, 0.70	911	Yes (~100 PPI)	*Nano Lett.* 17, 1319-1325 (2017)
	400 nm, 130 fs, 1 kHz	CdSe/Cd$_x$Zn$_{1-x}$Se/ ZnSe$_{0.5}$S$_{0.5}$/ZnS CQDs	1D DFB	2.1	627, <0.20	>3185	No	*Science* 365, 672-675 (2019)
	400 nm, 200 fs, 1 kHz	CdSe/CdS/ZnS CQDs	1D DFB	20.2	621, /	/	No	*Science China Information Sciences* 63 (2020)
	450 nm, 340 fs, 10 kHz	CdSe/CdS/ZnS CQDs	Plasmonic lattice	120-500	623-646, ~0.60	~1050	No	*ACS Nano* 14, 5223-5232 (2020)
	530 nm, 200 fs, 20 kHz	CdSe/CdZnS CQDs	BIC	36	648, 0.25	2590	No	*Nano Lett.* 20, 6005-6011 (2020)
	400 nm, 35 fs, 1 kHz	CdSe/CdS CQDs	Plasmonic lattice	30	633, 0.40	1583	No	*ACS Nano* 14, 3426-3433 (2020)
	400 nm, 130 fs, 1 kHz	CdSe/Cd$_x$Zn$_{1-x}$Se/ ZnS$_{0.5}$S$_{0.5}$/ZnS CQDs	1D DFB	5.5	631, <0.20	> 3160	No	*Nat. Commun.* 11, 271 (2020)
	1030 nm, 300 fs, 10 kHz	PbS CQDs	1D DFB	385	1650, 1.23	1341	No	*Advanced Materials* 34 (2021)
	1030 nm, 300 fs, 50 kHz	PbS CQDs	1D DFB	430	1650, 2.00	825	No	*Nat. Photonics* 15, 738-742 (2021)
	400 nm, 190 fs, 10 kHz	CdSe/ZnS CQDs	2D PhC	217	588-612, 0.33	~1800	No	*Nanophotonics* 12, 3257-3265 (2023)
	400 nm, 110 fs, 1 kHz	CdSe/CdS CQDs	1D DFB	12	628-634, 0.20	~3160	No	*ACS Photonics* 4, 2446-2452 (2017)
ns/sub-ns pulse	532 nm, 7 ns, 938 Hz			270			No	
	355 nm, 5 ns, 10 Hz	CdSe/ZnS CQDs	2D DFB	4000	610-640, ~4.00	~160	No	*Appl. Phys. Lett. 99, 241103 (2011)*
	532 nm, 8 ns, 1 kHz	CdSe/ZnCdS CQDs	1D DFB	250	608, <0.26	>2340	No	*Appl. Phys. Lett. 103, 171104 (2013)*
	355 nm, 5 ns, 10 Hz	CdSe/ZnS CQDs	1D DFB	500	610, 0.30	2033	No	*Opt. Express* 22, 7308-7319 (2014)
	355 nm, 5 ns, 10 Hz	CdSe/ZnS CQDs	1D DFB	372	600-618, ~0.60	~1000	No	*Appl. Phys. Lett. 104, 141108 (2014)*
	532 nm, 0.4 ns, 1 kHz	CdSe/ZnCdS CQDs	1D DFB	120 (red)	610, <1	>610	No	*Opt. Express 22, 18800-18806 (2014)*
	355 nm, 0.4 ns, 1 kHz	CdSe/ZnCdS CQDs	1D DFB	280 (green)<br>330 (blue)	575, <1<br>460, <1	>575<br>>460	No	
	532 nm, 0.4 ns, 1 kHz	CdSe/CdS/ZnS CQDs	2D PhC	300	622, /	/	No	*Nanoscale* 8, 6571-6576 (2016)
	532 nm, 0.4 ns, 1 kHz	CdSe/CdS/ZnS CQDs	2D PhC	1000	624, ~1.00	~624	No	*Opt. Express 25, 32919-32930 (2017)*
	532 nm, 0.3 ns, 1 kHz	CdSe/CdS/ZnS CQDs	1D DFB	360	613-623, ~0.60	~1030	No	*Nanoscale* 10, 22745-22749 (2018)
	343 nm, 1 ns, 800 Hz	CdSe-based CQDs	2D PhC	26.5	629-630, <0.2	> 3150	No	*Opt. Express* 29, 15145 (2021)
	355 nm, 5 ns, 1.25 kHz	CdSe/CdS CQDs	BIC	54	626, ~1.60	~390	No	*Nano Lett.* 21, 9754-9760 (2021)
	355 nm, 7 ns, 100 Hz	CdS bulk nanocrystals	2D PhC	98	516, 0.60	860	No	*Nature Nanotechnology 18,1423–1429 (2023)*
	355 nm, 0.3 ns, 100 Hz	CdZnSe/ZnSe/Zn$_x$Cd$_{1-x}$S CQDs	CBR	17	637, 0.37	1723	Yes (~2100 PPI)	This work
cw/quasi-cw	444 nm	CdSe CQWs	VCSEL	440 W cm^{-2}	530, 0.85	624	No	*Nature Nanotechnology* 9, 891-895 (2014)
	442 nm, 2.5 μs, 30 Hz	CdSe/CdS/ZnS CQDs	2D DFB	52 kW cm^{-2}	643, 0.70	919	No	*Nat. Commun.* 6, 8694 (2015).
	442 nm, 75 ms, 10 Hz	CdSe/CdS CQDs	2D DFB	6.4 kW cm^{-2}	639, ~1.00	~639	No	*Nature* 544, 75-79 (2017).

Supplementary Table 2. Comparison among the reported working stability of CQD and CQW lasers at room temperature

Pump source		Gain medium	Cavity structure	$P_{th,las}$ (μJ cm^{-2})	P_{test} (μJ cm^{-2})	Maximal operation duration (hrs)	Maximal T_{las}	Reference
fs pulse	400 nm, 150 fs, 1 kHz	CdS/CdZnS/ZnS CQDs	1D DFB	77	/	1.5	5.4×10^6	*Adv. Funct. Mater.* 22, 337-344 (2012)
	400 nm, 120 fs, 1 kHz	CdSe/CdS@$Cd_{1-x}Zn_xS$ CQWs	WGM	188	752	3	1.08×10^7	*Adv. Funct. Mater.* 30, 1907417 (2020)
	400 nm, 100 fs, 1 kHz	CdSe/CdS CQWs	VCSEL	1.1	3.5	4	1.44×10^7	*Adv. Optical Mater.* 8, 1901615 (2020)
	400 nm, 40 fs, 10 kHz	CdSe/ZnS CQDs	WGM	~100	~110	4.5	1.62×10^8	*Light Sci. Appl.* 10:60 (2021)
	1030 nm, 300 fs, 50 kHz	PbS CQDs	1D DFB	430	3000	1	1.8×10^8	*Nature Photonics* 15, 738–742 (2021)
	400 nm, 100 fs, 1 kHz	CdSe/CdS@ZnO CQDs	VCSEL	3.3	4.0	5	1.8×10^7	*Chem. Eng. J.* 428, 131159 (2022)
	1030 nm, 300 fs, 10 kHz	PbS CQDs	1D DFB	385	/	5	1.8×10^8	*Adv. Mater.* 34, 2107532 (2022)
	1030 nm, 300 fs, 10 kHz	PbS/PbSSe CQDs	1D DFB	160	/	8	2.88×10^8	*Adv. Mater.* 35, 2207678 (2023)
	450 nm, 120 fs, 1 kHz	CdS bulk nanocrystals	2D PhC	98	/	0.33	1.2×10^6	*Nature Nanotechnology* 18,1423–1429 (2023)
	400 nm, 200 fs	ZnCdSe/ZnSe/ZnS CQDs	WGM	22	/	/	$\sim 3 \times 10^7$	*Nano Lett.* 23, 437-443 (2023)
ns/sub-ns pulse	532 nm, 1 ns, 60 Hz	CdSe/CdS@$Cd_{1-x}Zn_xS$ CQWs	WGM	3.26	32.6	1.67	3.6×10^5	*Adv. Mater.* 34, 2108884 (2022)
	532 nm, 10 ns, 1 kHz	CdSe/CdS CQDs	F-P	7000	7000	9	3.24×10^7	*Light Sci. Appl.* 11, 275 (2022)
	532 nm, 1 ns, 60 Hz	CdSe/$Cd_{1-x}Zn_xS$ CQWs	WGM	27.7	277	2	4.3×10^5	*Laser Photonics Rev.* 17, 2200849 (2023)
	355 nm, 0.3 ns, 100 Hz	CdZnSe/ZnSe/$Zn_xCd_{1-x}S$ CQDs	VCSEL	56	110	200	7.2×10^7	This work
			CBR	17	38	1007	3.63×10^8	
